# Culturally adapting the Mediterranean Diet pattern – a way of promoting more ‘sustainable’ dietary change?

**DOI:** 10.1017/S0007114522001945

**Published:** 2022-08-28

**Authors:** Jayne Woodside, Ian S. Young, Michelle C. McKinley

**Affiliations:** 1 Centre for Public Health, Institute for Clinical Science A, Grosvenor Road, Belfast, Queen’s University Belfast, Belfast BT12 6BJ, UK; 2 Institute for Global Food Security, Queen’s University Belfast, Belfast, UK

**Keywords:** Mediterranean diet, Dietary pattern, Non-communicable disease risk, Cultural adaptation

## Abstract

Average diet quality is low in the UK and is socioeconomically patterned, contributing to the risk of non-communicable disease and poor health. Achieving meaningful dietary change in the long term is challenging, with intervention required on a number of different levels which reflect the multiple determinants of dietary choice. Dietary patterns have been identified which contribute positively to health outcomes; one of these is the Mediterranean diet (MD) which has been demonstrated to be associated with reduced non-communicable disease risk. Most research exploring the health benefits of the MD has been conducted in Mediterranean regions but, increasingly, research is also being conducted in non-Mediterranean regions. The MD is a dietary pattern that could have positive impacts on both health and environmental outcomes, while being palatable, appetising and acceptable. In this review, we consider the studies that have explored transferability of the MD. To achieve long-term dietary change towards a MD, it is likely that the dietary pattern will have to be culturally adapted, yet preserving the core health-promoting elements and nutritional composition, while considering the food system transition required to support changes at population level. Population-specific barriers need to be identified and ways sought to overcome these barriers, for example, key food availability and cost. This should follow a formal cultural adaptation framework. Such an approach is likely to enhance the extent of adherence in the longer term, thus having an impact on population health.

## Diet quality, non-communicable disease risk and behaviour change

Poor diet quality is dominant within the UK population^(1)^ and is associated with increased risk for non-communicable disease (NCD) such as CVD and type 2 diabetes (T2D)^(2)^. Latest analyses from the National Diet and Nutrition Survey suggest that there have been few major improvements in diet quality in recent years. For example, based on data from 2016 to 2019, only 33 % of adults (19–64 years), 40 % of older adults (65–74 years), 27 % of older adults aged 75 years and over and 12 % of 11–18 year olds met the 5 A Day fruit and vegetable (FV) recommendation^(3)^. These latest National Diet and Nutrition Survey figures also demonstrate that FV intake has changed little since 2008; adult intake remains at around 4 portions/d and children (11–18 years) around 3/d. Alongside concerns about diet quality and its association with health, there is an increasing concern about the impact of dietary choices on planetary health and how to encourage the adoption of dietary patterns that will have a lower impact on environmental outcomes^(4)^. For example, the EAT-Lancet report emphasises the need to transform to a universal healthy reference diet, which will be largely plant based, by 2050^(5)^. Achieving this change will require significant food system transformation and policymakers and research funders are becoming increasingly active in this area^(6,7)^. Globally, the WHO Sustainable Development Goals encourage the transformation of the food system^(8)^. Nationally, the National Food Strategy^(9)^ suggested the importance of food system transformation, with socio-economic inequalities, trade issues, links between diet quality and health and obesity being key foci and potential solutions suggested. The government white paper response is awaited. The devolved nations are following similar approaches regarding strategies and policy development (e.g. in Northern Ireland^(10)^). Increasingly, it is accepted that to achieve change towards healthy populations and environments will require a systems approach to capture the complexity and interconnectedness of the components of the food system^(7)^.

**Fig. 1.**
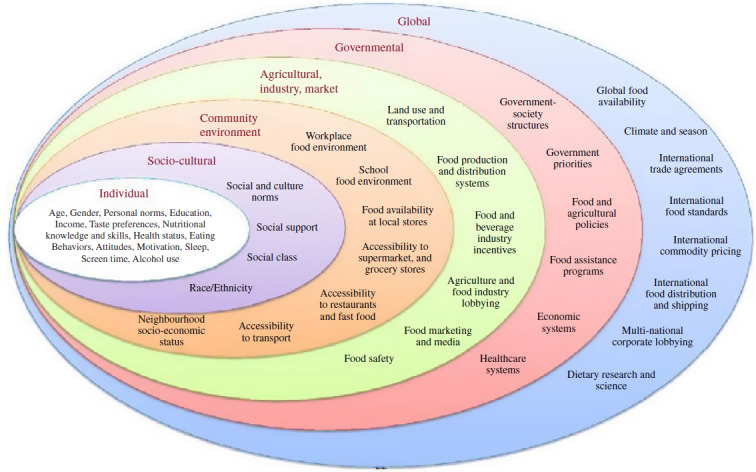
Barriers and opportunities for healthy eating. Reproduced with permission from^(15)^.

The invitation to complete this Horizons article follows a review published in this journal in 2013; that review focussed on FV, the evidence base for health benefits and how to increase intake^(11)^. At that point, it was noted that population levels of FV intake in the UK were low and that interventions to increase intake were challenging in terms of changing behaviour and sustaining it in the long term^(11)^. One of the systematic reviews included suggested a need to better understand factors influencing FV consumption, including economic, social and environmental factors that affect both food availability and the ability of an individual to make healthy choices, and the barriers to making changes towards increased FV intake^(12)^. A further systematic review considered that there was a lack of clear linkage between behavioural theories and constructs and the interventions being developed to encourage increased intake^(13)^. The authors recommended combining behavioural interventions with social marketing, behavioural economics approaches and technology-based behaviour change models in future studies, and always including both process and outcome evaluation^(13)^. The conclusion of the review was that achieving a sustained increase in FV intake is challenging and would require the development and testing of further novel multi-faceted intervention approaches. What has emerged more clearly since that time is that achieving long-term behaviour change requires intervention on a number of different levels in-line with the multiple determinants of dietary choice including individual, sociocultural, community, agricultural, industry, governmental and global levels. Potential policy strategies can be applied across a range of different domains and sectors^(14)^, as outlined in Fig. 1^(15)^, and reduction of health inequalities as a result of such strategies consistently needs to be considered. It has been suggested that a road map for sustainable behaviour change will be more likely to succeed if efforts are harmonised across different settings (e.g. schools, workplaces, hospitals), at multiple levels (e.g. individual, household and community) and involve a diverse set of stakeholders (e.g. health care professionals, industry, policymakers)^(16)^. The impact of different policy actions to improve population dietary patterns has been systematically reviewed^(17)^ and the need to include availability considerations recently highlighted^(18)^. Examples of availability considerations will require food environment modifications and include a change to the variety of options (their number or range) available to consumers in absolute or relative terms. This can occur across a broad spectrum of settings in countries (such as limiting the availability of outlets selling higher strength alcohol), neighbourhoods (such as reducing the density of unhealthier food outlets) or retail premises, such as a shop, café, restaurant or bar, in private and public sector settings (e.g. canteens and vending) ^(18)^.

### Dietary patterns and health

Alongside this improved understanding of the complexity of achieving sustained behaviour change, diet and health research has shifted to include a more holistic consideration of the complexities of diet, via dietary pattern analysis, alongside evaluation of the specific roles of individual nutrients in terms of NCD risk (e.g. via single micronutrient supplementation trials)^(19)^. Dietary pattern analyses can take either *a priori* or *a posteriori* approaches^(20)^. *A posteriori* approaches aggregate specific food items or food groups on the basis of the extent to which those items or groups correlate with each other, thus identifying common underlying patterns of food consumption, using approaches such as principal component or cluster analysis^(20)^.

An alternative is to use our knowledge of the relationship between dietary components and health to derive composite *a priori* scores which can be used to measure adherence to a particular dietary pattern; the resulting scores can then be associated with health outcomes or used to measure change within dietary interventions^(21)^. Since *a posteriori* approaches derive dietary patterns from within an individual data set, they are population-dependent, whereas a range of *a priori* dietary pattern scores, related to overall nutritional quality and associated with a range of health outcomes, have been developed^(22)^ and applied independently of the population under study.

### The Mediterranean Diet

**Fig. 2.**
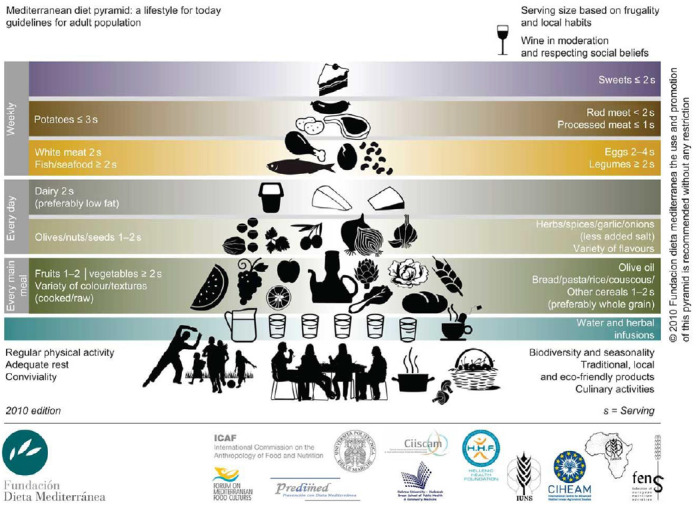
Mediterranean diet PYRAMID. Reproduced with permission from^(23)^.

The Mediterranean diet (MD) is widely accepted as an example of a healthy dietary pattern. It reflects the diet traditionally consumed by those living in Mediterranean regions, characterised by a high consumption of fruits, vegetables, wholegrains, legumes, nuts and seeds, moderate amounts of dairy products, moderate amounts of red wine, low to moderate amounts of fish and poultry and low amounts of red meat, with olive oil as the principal source of fat^(23)^. A representation of the MD pattern in the form of a pyramid is shown in Fig. 2^(23)^ and a range of *a priori* scores to assess adherence have been developed^(24)^. UNESCO has recognised the MD as an Intangible Cultural Heritage of Humanity^(25)^.

Increased adherence to a MD has been associated with greater longevity^(26)^, a lower prevalence of several chronic diseases, including CVD and cancer, better management of T2D^(26–29)^ and reduced risk of cognitive decline^(30)^. Adherence to a MD has also been associated with improvements in risk factors for both CVD and T2D, including blood pressure, lipids, biomarkers of inflammation and insulin resistance^(31)^. Dietary intervention studies with hard clinical outcomes are rare in nutrition science; but such studies have been conducted using a MD-style intervention. In a population at increased risk of CVD, the *Prevención con Dieta Mediterránea* (PREDIMED) trial demonstrated that adoption of a MD led to an approximate 35 % (hazard ratio 0·65; 95 %CI 0·50, 0·85) reduction in CVD incidence and a 52 % (hazard ratio 0·48 (95 % CI 0·27, 0·86) reduction in T2DM within the MD intervention arm (supplemented with nuts or extra virgin olive oil) compared with the low-fat diet control arm^(32,33)^.

Given these proposed health benefits, the MD has been suggested as an appealing way of promoting healthy eating^(34)^, potentially without increased cost^(35)^. The EU has endorsed the MD, encouraging promotion of ‘healthy eating, emphasising health-promoting diets, such as the MD’^(36)^. In terms of environmental outcomes, and the need to move towards a more plant-based dietary pattern, the MD is also potentially a useful model. The MD is plant-rich but does not exclude animal-sourced foods and instead moderates them – therefore this dietary pattern may have positive effects on both environmental and health outcomes. The sustainability of the MD has been demonstrated in Mediterranean regions^(37–39)^; however, sustainability in non-Mediterranean regions and impact of climate change on future food production is less explored^(40,41)^. Thus, there is increasing focus on both nutritional and environmental impacts of dietary patterns including the MD pattern, but this still needs to be considered in regions outside of the Mediterranean^(42)^.

### Transferability of the MD to non-Mediterranean regions

Many observational studies examining the association between MD adherence and clinical outcomes have been based in Mediterranean populations, but the association has also been demonstrated in non-Mediterranean populations^(43)^.

Given the proposed health benefits of the MD, encouraging non-Mediterranean populations to increase adherence to such a dietary pattern would potentially improve NCD, but also address environmental priorities. Transferability and adoption of a MD beyond the Mediterranean region does, however, present a number of challenges.

Firstly, there is marked variation in MD adherence and in the key foods consumed within the pattern depending on the country in which it is being assessed^(44)^. Specifically, accessibility of MD foods, such as extra-virgin olive oil, and barriers to adoption due to culture, religion, traditional cooking practices and economic burden, need to be addressed in interventions aiming to increase MD adherence in non-Mediterranean populations^(45,46)^. There may be cultural barriers against changing current food preferences^(47)^ thus supporting the need to make dietary advice culturally appropriate^(48,49)^. Incorporating foods that are culturally acceptable, locally produced and accessible, but with a similar nutritional profile to those prominent in a MD, may further encourage MD adherence in non-Med countries, and such dietary pattern adaptations which focus on locally produced and acceptable foods have been studied^(49,50)^. Regional adaptations are also important from an environmental and cost perspective, which are issues that have been raised when exploring barriers to MD in non-Mediterranean populations^(47,51)^. Cost of included foods and how this drives choice may be particularly important; the association between MD adherence and health outcomes has recently been demonstrated to depend on socio-economic status. In the Moli-sani study in groups with similar MD dietary adherence scores, diet-related disparities in CVD risk persisted across socio-economic groups. It was suggested this may be related to the nutritional value of the foods selected, even within these similar overall MD adherence scores, which may be related to cost^(52)^. Selection of culturally appropriate and affordable foods may decrease socio-economic disparities when implementing a MD in a non-Mediterranean country.

A further issue is that previously conducted clinical trials have generally achieved successful dietary change toward a MD using resource-intensive interventions, mainly delivered by health professionals and usually in parallel with food provision^(33,53,54)^. Although effective, such strategies are not practical or cost effective when considering a population-level approach to behaviour change. Evaluation of less intensive modes of delivery that can be both effective and cost-effective at a population level is needed in order to realise the benefits of the MD.

Preliminary work conducted by our group has demonstrated increased MD adherence in those at high CVD risk^(53,55,56)^, highlighting the potential for translation to non-Mediterranean regions, and has also evaluated less intensive modes of delivery to achieve dietary change. Our work builds on that conducted by others in the UK exploring barriers and facilitators to increasing adherence to a MD in different population groups, and then guiding intervention development^(51,57–61)^. The Trial to Encourage Adoption and Maintenance of a MEditerranean Diet study (TEAM-MED) was a pilot trial implemented in Northern Ireland (NI), aiming to explore methods of increasing MD adoption in a non-Mediterranean population at high risk of CVD. This 12-month pilot study explored the feasibility of a peer support intervention *v*. a previously tested dietitian-led intervention^(47,55,62–64)^ to encourage MD behaviour change, following MRC guidance for developing and evaluating complex interventions^(65)^. For all participants, increases in MDS were observed over 12 months (*P* < 0·001), and there were improvements in BMI, HbA1c levels, systolic and diastolic blood pressure in the population as a whole, demonstrating that a non-Mediterranean adult population at high CVD risk can make dietary behaviour change towards a MD over a 12-month period^(55)^. As already established groups may have greater social cohesion and engagement compared with members in newly-formed groups and the leveraging of existing social networks between members may enhance the effectiveness of peer support strategies to further encourage behaviour change, the TEAM-MED EXTEND feasibility study^(56)^ then evaluated the feasibility of a peer support intervention to encourage adoption and maintenance of a MD in *established* community groups. Feasibility issues around working were observed within established community groups, but an increase in MDS was observed in all intervention groups at 6 and 12 months, therefore suggesting the adoption of components of a MD pattern.

While these findings indicate that behaviour change towards a MD is achievable in a non-Mediterranean population for up to 1 year, there is still much to learn regarding the most cost-effective approaches to support MD behaviour change^(66)^, and particularly, interventions to support longer-term maintenance of newly adopted MD behaviours in non-Mediterranean countries.

### Other uncertainties regarding the MD that remain to be addressed by nutritional science

It has been highlighted already that the flexibility and evolution of the MD pattern present some issues with regards to assessing the association with health outcomes, but also in terms of defining the pattern, the key elements of it and what components have to be included to maximise the health benefits. Available scoring systems reflect this heterogeneity^(67)^. Dominant MD researchers have explored this and suggest that transferability can incorporate flexibility but stress the need to incorporate all traditional components and also to substantially reduce or even completely avoid elements which are fully in opposition to the concept of the traditional MD^(68)^. They also point to some of the systematic reviews of the health benefits of the MD where incorporation of at least two of nine MD components was then defined as being a MD, which authors point out lacks specificity^(68)^. A similar discussion paper, although focussed on T2D, stresses the need to report the specific descriptions of dietary targets within MD interventions rather than general recommendations, as that will help assessment of how closely these interventions align with the traditional MD^(69)^. Authors highlight that the variety of vegetables and whole grains, which are key features, are often omitted in such descriptions; they also highlight that prudent dietary patterns that are identified through *a posteriori* analyses or that are developed as being consistent with national dietary guidelines will share similarities with the MD, but will not be the same^(69)^. It is acknowledged that different interpretations of the MD are not surprising, given that the dietary pattern is a product of different countries with different cultures and social, economic and historical contexts across time in the Mediterranean region and that it is evolving over time. Even the clinical trials of MD have differed quite markedly in the details of their interventions and it has been suggested that neither the PREDIMED nor the Lyon Diet Heart studies can be considered strictly traditional MD interventions. Nevertheless, researchers in the field have called for greater consistency and accuracy in the reporting and operationalisation of a MD and that reference should be made to a Mediterranean-style diet to preserve the uniqueness of the traditional pattern^(69)^.

The most recent American Heart Association/American College of Cardiology recommendations include the MD as an example of a healthy dietary pattern that achieves adherence to their guidelines, although the strength of evidence was overall rated as low^(70–72)^. This low evidence rating was given due to two factors: firstly, the cross-cultural variability in the application of the MD which then limits the ability to calculate precise estimates of the association with specific benefits, for example, risk factor reduction; and, secondly, because the PREDIMED trial had two active interventions which included the provision of food supplements, leading to concerns, as already mentioned, over whether the trial really tested the impact of MD adherence on outcomes, although this assertion has been strongly refuted by the PREDIMED authors^(68,73,74)^. These two factors are raised repeatedly in the literature regarding the testing of MD interventions, that is, if adaptations are made for cultural/food availability/promoting adherence reasons does that still mean a MD is being tested, and the fact that these adaptations and the flexibility in the dietary pattern then have implications for future evidence synthesis.

A further critical appraisal of the MD for non-Med populations makes similar points, highlighting the heterogeneity in, for example, vegetable types but also preparation methods that may influence impact on health outcomes, and also stresses the key importance of extra-virgin olive oil to the pattern^(75)^. So the nature of the food itself is important, alongside variation in food source, production methods and preparation. The other components of a Mediterranean lifestyle, which are rarely considered in dietary interventions to date, such as the eating of meals together, lengthy meal times, the habit of having a siesta in the middle of the day, and the potential confounding exposure to sunlight in Mediterranean regions and resulting impact on vitamin D status, could also be important^(75)^.

In summary, these issues represent challenges and opportunities for innovative nutrition research. These include a better understanding of the key components of a MD in terms of health promotion, how the diet pattern can be adapted while retaining a similar nutritional profile, and how best to describe adherence to the diet (e.g. scoring systems and via objective biomarkers) so that links with health outcomes can be better assessed, particularly during evidence synthesis. The mechanisms behind the health benefits would benefit from further exploration, as would the additive effects of the broader elements of the Mediterranean lifestyle beyond diet. The implications of closer adherence to the dietary pattern on costs, and to what extent this is a pattern that can be adopted by all, regardless of income and food literacy, needs to be considered. Finally, the impact of a food system transition leading to greater population adherence to a MD on sustainability outcomes, given local food production and supply contexts, still needs to be explored.

### Cultural adaptation to improve transferability of the MD pattern

In our recent dietary intervention studies^(55,56)^, whilst we assessed barriers and facilitators to adopting a MD pattern in the local setting^(47,63)^, we did not formally culturally adapt the dietary intervention. Cultural adaptation may help promote longer-term adherence on a population level, although preserving the core elements of the MD pattern and its nutritional composition will be important, as previously suggested^(68)^. These core elements have been suggested to include high consumption of olive oil, legumes, cereals, FV, moderate to high consumption of fish, low consumption of meat and meat products, and moderate consumption of dairy products, mostly as cheese and yogurt and wine^(68)^. The MD pattern could be adapted to utilise locally produced and available foods where possible, producing a pattern that has the MD health benefits, addresses inadequacies in dietary intake, and is culturally acceptable and accessible to a non-Mediterranean population. Such a dietary pattern would resemble the MD, but with food substitutions as appropriate (e.g. rapeseed oil for olive oil, use of MUFA-rich spreads for populations where oil use is less familiar, local fish and wholegrain sources). The impact of these substitutions on overall nutritional compositions needs to be assessed as the use of olive oil is central to the traditional MD^(68,76)^.

The adaptation of the MD has been explored in other global regions. In Australia, George *et al.* developed an MD model complying with the principles of the traditional MD and applied in a multi-ethnic context^(77)^. Firstly, the nutrient profile of a MD was identified from previous MD interventions and this was then used to identify key food-based components, with these two steps being combined to develop a 2-week meal plan. Potential barriers to translating the MD into the Australian population were then identified using the application of a SWOT framework, with appropriate strategies embedded into the intervention to address these perceived barriers^(77)^. The intention is to test this MD model in two clinical settings – patients with NAFLD and within the secondary prevention of CHD^(77)^, and early data from these trials are starting to emerge with intermediate outcomes^(78,79)^.

The MD intervention studies that have been conducted so far in Australia have been reviewed^(80)^ and acknowledge that the MD represents a dietary pattern that could address both environmental and health concerns. However, authors do note significant heterogeneity in the eight identified studies that have claimed they tested a MD pattern in terms of the dietary protocols and a lack of prescriptive interpretation of a MD across the studies. This makes the interpretation of both the efficacy and assessing adherence to the dietary pattern difficult; determining whether this heterogeneity is important in terms of the health outcomes will be challenging but also important.

Key approaches to implementing successful MD intervention in a non-Mediterranean setting have been suggested, based on three intervention studies and largely considering the Australian context^(81)^. These include it being dietitian-led, with accompanying written resources, inclusion of food hampers, regular contact with healthcare professionals or volunteers/others making dietary change, for example, at cooking classes and provision of recipes. These are relatively high-intensity approaches that may not be cost-effective for roll-out at scale. Considering population-wide adoption, and, as suggested earlier, a multi-sectoral approach is called for with the collaboration and commitment of food industry, food retailers, regulatory bodies, policy makers and health professionals. The framework needs to consider a socio-ecological model to accommodate all settings and the dietary pattern itself must be cost effective in terms of health outcomes, but also affordable for the consumers and, importantly, culturally acceptable^(81)^.

Perceived enablers or barriers toward adherence have also been explored in the Australian setting using a self-administered online questionnaire, which included questions aligned with the Theory of Planned Behaviour ^(82)^. Barriers and enablers toward adherence to MD were grouped under the three core constructs of the Theory of Planned Behaviour: attitudes (suitability, taste, restrictive and food waste); social norms (food culture); and perceived behavioural control (PBC) (motivation, affordability, time/effort, food access, knowledge, food outlets, natural conditions, cooking skills). PBC emerged as the most prominent construct influencing intention to follow a MD. Perceived health benefits (*n* 445; 76·5 %) and improved diet quality (*n* 224; 38·5 %) were identified as major advantages. In contrast, the need for dietary adherence (*n* 147; 39·7 %) was perceived as an important disadvantage. Authors suggested that future MD interventions should consider adopting strategies aimed at improving self-efficacy to reduce self-perceived barriers and facilitate dietary adherence^(82)^.

Although the greatest volume of literature exploring MD transferability and adaptation has focussed on the UK and Australia, the MD has been adapted and tested in other countries^(83,84)^. For example, del Campo *et al.*^(83)^ explored the feasibility and acceptability of a previously tested and evidence-informed lifestyle intervention to reduce CVD risk in low-income Hispanic American women. The dietary pattern was MD style but was adapted for the study population in a formative phase; intervention engagement and acceptability were high and there was an improvement in self-reported dietary behaviours. The possibility of an adapted MD pattern that is adaptable, applicable and sustainable within the Chilean context and has the potential to address the current trend of chronic diseases in the country, taking account of the socio-cultural reality of the community or population, has also been discussed^(84)^.

#### Nordic diet

The New Nordic diet is an example of a dietary pattern that shares many characteristics with the MD but comprises foods traditionally sourced in Denmark, Finland, Iceland, Norway and Sweden^(50)^. Staple components of the New Nordic diet include berries and fruits, fatty fish (herring, mackerel and salmon), lean fish, legumes, vegetables (cabbage and root vegetables) and whole-grain cereals (barley, oats and rye). A notable point of difference compared with the MD is the use of rapeseed (canola) oil instead of olive oil^(50)^. The Nordic diet is predominantly plant-based and locally sourced, thus ensuring more environmentally friendly production with reduced waste when consumed within the Nordic region^(50)^. Such a dietary pattern can, therefore, be considered to be culturally adapted. In terms of nutrient composition, the Nordic diet is similar to the MD, except in terms of the primary fat source as already discussed^(85)^.

Research has demonstrated the health-promoting properties of the New Nordic diet, including protective effects against CVD and T2D, although it has been less intensively researched compared with the MD and has focussed on cardiovascular risk factors rather than hard clinical outcomes^(86,87)^.

WHO/Europe conducted a new Health Evidence Network Synthesis Report exploring the implementation and effectiveness of policies based on the New Nordic (and Mediterranean) diets^(50)^. The report found that a total of fifteen countries in the European Region currently recommend or implement policies based on the New Nordic or MD, emphasizing the health benefits and – in some cases – the cultural significance of these diets. In addition, the Nordic countries in particular have demonstrated collaborative policy-making through between-country initiatives^(50)^. However, the report found less evidence of policy development than expected and limited evidence that impact is being routinely evaluated^(50)^. Nevertheless, such adaptation and engagement with policymakers, including the previously highlighted multi-sectoral approach, should be considered as a key example when culturally adapting a healthy dietary pattern in other settings^(50)^.

### Cultural adaptations of other lifestyle interventions

Cultural adaptation of evidence-based lifestyle interventions is therefore likely to be a necessary action when encouraging them to be adopted by a new population^(88)^. It implies modifications meant to improve an intervention’s fit in a specific context and for sub-cultural groups, considering the unique cultural values, beliefs, socio-economic status and environment of these populations, while preserving the fidelity of the core elements of the intervention^(89)^. Cultural adaptation also has the potential to improve the reach, engagement, effectiveness and sustainability of programs while reducing health inequalities^(90)^. Improving health behaviours may be less effective if investigators disregard the need for cultural adaptation or inadequately adapt interventions^(91)^.

The potential of culturally appropriate dietary advice has recently been highlighted, although not in relation to the MD^(49)^. Providing more inclusive culturally or socially relevant nutrition advice may promote behaviour change in the longer term and account for personal dietary preferences or patterns. Example studies include testing the traditional Mexican diet compared with a more Americanised diet among first- and second-generation women of Mexican descent, which found that the traditional diet modestly improved insulin sensitivity^(92)^, and avoiding culturally discordant advice to Asian Indian patients^(93)^. Even here it is important to be sensitive to differences within culturally similar groups in terms of dietary preferences, for example, by age^(49)^, whilst different levels of adherence to MD advice have also been associated with partners’ adherence to MD, suggesting a mediation by household members’ support and, in particular, family function^(94)^. Other examples of cultural adaption have occurred in terms of weight-loss interventions in children^(95)^, lifestyle management in relation to T2D^(96–99)^, school-based smoking cessation^(100)^ and weight management in fathers^(101)^.

An examination of cultural adaptation of lifestyle interventions for the prevention of T2D in South Asian populations in Europe^(97)^ agreed that a structured approach to understand and advance cultural adaptation is important to develop effective health promotion programmes to tackle inequities in health. The authors acknowledge that their findings do not fully unpack ‘how’ these interventions work, for ‘whom’ and in ‘what contexts’, but they do assist in proposing a structured approach to undertake further ‘sense-making’ of existing approaches to adaptation, and also highlight some of the key contextual considerations which influence the outcomes of these interventions^(97)^. The authors suggest greater consideration of how adaptations fit with heterogeneous and intersecting population characteristics; how intervention design can safeguard sustainability beyond any investigative phase, ultimately becoming embedded in established health service or community structures; and lastly, what broader contextual influences may limit participant capacity for behaviour change and if there is any way to address or mitigate these influences^(97)^.

A range of studies have explored the impact of cultural adaptations on the effectiveness of various interventions, and those including diet studies, targeting smoking cessation and/or physical activity have been systematically reviewed^(102)^. Studies have tended to focus on adaptation targeted to ethnic minority populations living in a high-income society. Authors found that culturally targeted behavioural interventions may be more effective if cultural adaptations are implemented as a package of adaptations, that the adaptation includes a consideration at family level, and where the adaptation results in a higher intensity of the intervention. They also concluded that more systematic experiments are needed in which the aim is to gain insight into the best mix of cultural adaptations among diverse populations in various settings, particularly outside of the USA, which is where such efforts have principally been focussed^(102)^.

A number of frameworks to culturally adapt evidence-based interventions have been suggested^(103–107)^. For example, the Framework to Report Adaptation and Modifications-Expanded (FRAME) provides a guide for conceptualising the different sorts of modifications that may be part of an adaptation and links together their process, nature and outcomes^(89,103)^. This framework draws upon a systematic review of frameworks and cultural adaptations of interventions in the USA and Latin, European and Asian countries^(89)^. FRAME uses eight different aspects that detail what adjustments have been made to the original version, where they were made, when they were made, who they were made by and why they were made^(89, 104)^.

Other tools and frameworks, for example, the Typology of Adaptation, Programme Theory of Adapted Health Promotion Interventions and RESET tool have been suggested to help develop more considered approaches to adapting interventions^(105)^. The RE-AIM (Reach, Effectiveness, Adoption, Implementation, Maintenance) framework has also been used^(106)^, incorporating quantitative (baseline characteristics, implementation metrics, effectiveness outcomes and costs) and qualitative (semi-structured interviews with stakeholders) data collection and mixed-method analysis. Barrera and Castro^(107)^ proposed a heuristic model for guiding cultural adaptations of evidence-based interventions and outlined stages for their development. The stages of adaptation development are (a) information gathering, (b) preliminary adaptation design, (c) preliminary adaptation tests and (d) adaptation refinement. These proposed stages therefore also combine quantitative and qualitative data to inform decisions about intervention adaptations.

Overall, although understanding of how to approach formal cultural adaptation of a MD is at an early stage, MD adaptation should follow an established framework and use mixed methods (exploring factors shown to influence behaviour change, such as taste, cost, availability, access, self-efficacy, perceptions, sustainability/environmental concerns, social norms and food culture), with Personal and Public Involvement/stakeholder input.

With regards specifically to nutrition, it has been questioned whether the MD pattern is truly the optimal healthy dietary pattern for a UK population^(43)^. To clarify this, it has been suggested that conducting a nutritional survey and generating *a posteriori* dietary patterns to reveal the local healthy dietary patterns which could then be compared with the original MD and differences between the two patterns identified could be a useful way to start cultural adaptation and identify important features^(75)^.

### Conclusion

Given the burden of NCD within the population, the association with diet quality, the observed stagnation in population dietary intake and the growing concerns about environmental impact of the current UK food system, we need a robust evidence base to underpin food, environment and public health policy.

Dietary patterns have gained prominence as a complementary approach to more traditional single nutrient research when exploring diet and health, and the MD pattern has been heralded as a dietary pattern that could have positive impacts on both health and environmental outcomes, while being palatable, appetising and acceptable. There are issues with transferability, specifically in terms of maintaining the key components of the diet/lifestyle pattern that has a demonstrated health benefit, but also culturally adapting the pattern and ensuring food system transition to promote population-level uptake without widening socio-economic inequalities, and, importantly, considering impact on environmental outcomes in different settings. These represent challenges that can be considered from an implementation science perspective but that will also require further innovative nutrition research.

We suggest that, when adapting the MD for a non-Mediterranean population, it is important to identify barriers to a MD and to culturally adapt the intervention, using established approaches that have been applied in other settings; this may include the substitution and use of locally produced, familiar foods. To implement population-level behaviour change in the long term will require multi-sectoral involvement and activity to achieve the sort of food system transformation required.

Despite these challenges, supporting adoption and maintenance of a healthy dietary pattern such as the MD, which has been shown to effectively reduce the risk of cardiovascular risk factors, CVD and T2D, should be a high public health priority for disease prevention.
